# Circulating Protein Biomarkers in Systemic Sclerosis Related Pulmonary Arterial Hypertension: A Review of Published Data

**DOI:** 10.3389/fmed.2018.00175

**Published:** 2018-06-06

**Authors:** Peter M. Hickey, Allan Lawrie, Robin Condliffe

**Affiliations:** ^1^Department of Infection, Immunity and Cardiovascular Disease, University of Sheffield, Sheffield, United Kingdom; ^2^Pulmonary Vascular Diseases Unit, Royal Hallamshire Hospital, Sheffield, United Kingdom

**Keywords:** systemic sclerosis, pulmonary arterial hypertension, protein, biomarkers, diagnosis

## Abstract

Pulmonary arterial hypertension (PAH) develops in 7–12% of patients with systemic sclerosis (SSc) and is associated with a 3 year survival of 52%. Early detection by screening is therefore recommended for all patients with SSc. Historically, screening has been performed using echocardiography and measurement of gas transfer. More recently the DETECT protocol, using a combination of biomarkers (including N-terminal pro-brain natriuretic peptide) and clinical parameters, has been developed. The optimal method of screening for PAH with high sensitivity and specificity is, however, not clear. Protein expression differences between different SSc disease phenotypes have been reported, and include alterations in concentration of NT-proBNP, endoglin, soluble vascular endothelial growth factor receptor 1, placenta growth factor, growth differentiation factor-15, vascular endothelial growth factor alpha, resistin-like molecule beta, and soluble thrombomodulin. This review summarizes the current knowledge of these protein changes in patients with SSc and PAH.

## Introduction

Pulmonary arterial hypertension (PAH) is a rare disease characterized by a progressive vasculopathy of the small pulmonary arteries leading to increased pulmonary vascular resistance and right ventricular afterload. This results in right ventricular failure and premature death. PAH may be idiopathic, heritable, related to various drugs and toxins or may be associated with a number of medical conditions including congenital systemic to pulmonary shunts, portal hypertension, HIV and connective tissue disease, most notably systemic sclerosis (SSc). Although these forms of PAH have similarities in underlying pathophysiology and treatment regime, it is increasingly recognized that there are differences in pathobiology, response to treatment and prognosis.

Between 7 and 12% of patients with SSc develop PAH (SSc-PAH) at some point in their disease course (1–4). Compared to idiopathic pulmonary arterial hypertension (IPAH), histological patterns in SSc-PAH show greater intra-individual variability, a relative absence of plexiform lesions, more prominence of intimal fibrosis and a much greater involvement of pulmonary venules (5). Prognosis is significantly worse than in other forms of PAH with median survival of 3 and 7.8 years in SSc-PAH and IPAH cohorts respectively (6). Response to treatment is generally felt to be worse in SSc-PAH than in IPAH (7).

The diagnosis of PAH can be challenging, due in part to the relatively low incidence of this condition, and in part due to the non-specific nature of the early symptoms. As a result, the diagnosis of PAH is often made at an advanced stage after a large proportion of the pulmonary vascular bed has been irreversibly obliterated by pathological vascular remodeling.

Given the progressive nature of vascular remodeling in PAH, interest in the outcome of patients detected early has led to investigation of alternative screening methods in patients with systemic sclerosis. It is thought that early diagnosis and treatment leads to a favorable prognosis (8, 9). Historically, screening for PAH in SSc has involved estimating systolic pulmonary arterial pressure at annual echocardiography ± the measurement of diffusion capacity of the lung for carbon monoxide (DL_CO_). The DETECT protocol was developed to provide an evidence-based multi-modality approach to screening patients with SSc for the presence of PAH. It combines variables from clinical examination, echocardiography, electrocardiography, pulmonary function tests and blood tests [uric acid and N-terminal pro-brain type natriuretic peptide (NT-proBNP)] to produce a score with high sensitivity (95%), but relatively low specificity (48%) for the presence of PAH (10). The DETECT protocol involves several steps which add complexity and in real-life clinical practice, may prove significantly more expensive and can lead to delay and even failure to refer for definitive investigations (11).

Abnormal concentrations of many candidate proteins have been shown in both the tissue and circulating compartments in patients with PAH. These proteins may reflect the underlying pulmonary vascular disease as well as the response of the right ventricle to the increased afterload. They may also reflect the underlying SSc rather than PAH. It is conceivable that a panel of protein biomarkers may have utility in identifying PAH in still asymptomatic patients with systemic sclerosis. This paper reviews the current published evidence base for altered protein biomarker concentrations in SSc-PAH.

## Methods

To identify suitable primary research articles on this topic, a literature search was conducted using Ovid Medline and PubMed. Keywords used were “Systemic sclerosis,” “Scleroderma,” “Pulmonary hypertension,” “PAH,” “Protein,” and “Biomarker.” Date of publication was limited from 1990 to present day. One hundred and forty eight publications were returned from this search.

We included studies identifying a cohort of patients diagnosed with systemic sclerosis with PAH with comparator groups including healthy volunteers (HV), systemic sclerosis without pulmonary hypertension (SSc-no PAH) and/or idiopathic PAH.

Studies were included if they reported data on differential protein expression between subgroups which were related to objective measurements of pulmonary hypertension.

### Protein biomarkers in SSc-PAH

**Figure 1:**
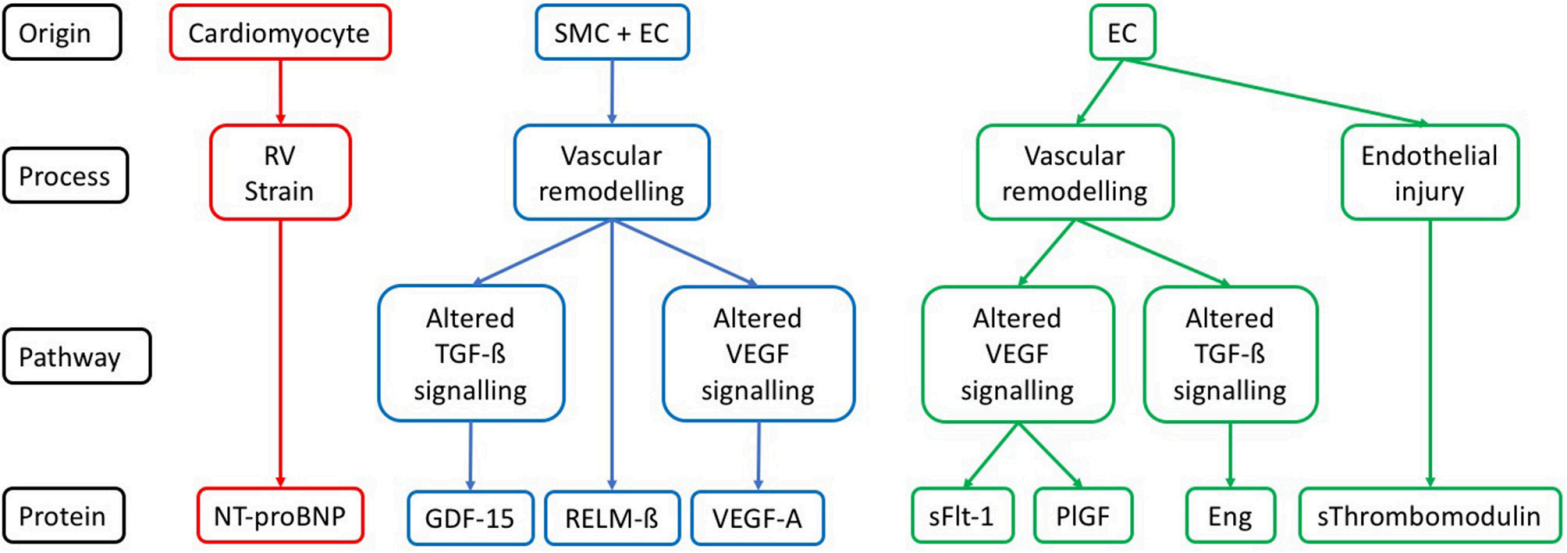
Cellular origin and pathways for each protein described in the context of SSc-PAH. Description of the likely origin of each protein, along with the pathophysiological process it has a role in. If a component of one of the pathways known to be relevant to pathogenesis of PAH then this is also given. SMC, vascular smooth muscle cell; EC, vascular endothelial cell; RV, right ventricle; TGF-ß, transforming growth factor beta; VEGF, vascular endothelial growth factor; NT-proBNP, N-terminal pro-brain natriuretic peptide; GDF-15, growth differentiation factor-15; RELM-ß, resistin-like molecule beta; VEGF-A, vascular endothelial growth factor A; sFLT-1, soluble vascular endothelial growth factor receptor 1; PlGF, placenta growth factor; Eng, endoglin; sThrombomodulin, soluble thrombomodulin.

The circulating protein biomarkers identified by the literature search are summarized in Table 1 and Figure 1.

**Table 1:** Summary of potential protein biomarkers in SSc-PAH.

**Protein**	**Comparison Groups**	**Number of Patients**	**Outcome**	**Correlations in SSc-PAH**	**Reference**
NT-proBNP	SSc-PAH vs. SSc SSc-PAH vs. SSc SSc-PAH vs. IPAH	109 329 98	Significantly higher in SSc-PAH vs. SSc. Sens 55.9%, Spec 95.1%. Correlated with invasive hemodynamics NT-proBNP superior to BNP for detection of PAH in SSc Significantly higher in SSc-PAH, correlated with hemodynamics and predicted survival in SSc-PAH group.	mPAP (*r* = 0.62; *p* < 0.0001) PVR (*r* = 0.81; *p* < 0.0001) CI (*r* = −0.58; *p* < 0.01) PVR (*r* = 0.54; *p* < 0.01)	(12) (13) (14)
Endoglin	SSc-PAH vs. SSc vs. HV	60	Serum levels significantly higher in SSc-PAH than control		(15)
sFLT-1	SSc-PAH vs. SSc	77	Plasma levels significantly higher in SSc-PAH and correlate with RVSP and inversely with DL_CO_. Possible predictor of PH progression.	RVSP (*r* = 0.32; *p* = 0.01) DL_CO_ (−0.29; *p* = 0.01)	(16)
PlGF	SSc-PAH vs. SSc	77	Plasma levels significantly higher in SSc-PAH. Correlates with severity of Raynaud's phenomenon and inversely with DL_CO_.	DL_CO_ (*r* = −0.031; *p* = 0.01)	(16)
VEGF-A	SSC-PAH vs. SSc vs. HV	53	Serum levels significantly higher in SSc-PAH than either SSc or HV. Levels correlate with echocardiographic sPAP, dyspnoea score and DL_CO_.	sPAP (*r* = 0.58; *p* < 0.01) DL_CO_ (*r* = −0.47; *p* < 0.01)	(17)
GDF-15	SSc-PAH vs. SSc	54	Plasma levels significantly higher in SSc-PAH, correlate with echocardiographic RVSP and circulating NT-proBNP. Discriminates between PH and non-PH.	RVSP (*r* = 0.56; *p* < 0.001)	(18)
RELM-ß	SSc-PAH vs. IPAH vs. HV	26	Tissue concentrations significantly higher in SSc-PAH than in IPAH or HV.		(19)
sThrombomodulin	SSc-PAH vs. SSc vs. HV	92	Significantly higher plasma levels in SSc-PAH compared to either SSc or HV.		(20)

*NT-proBNP, N-terminal pro-brain type natriuretic protein; sFLT-1, soluble vascular endothelial growth factor receptor 1; PlGF, placenta growth factor; VEGF-A, vascular endothelial growth factor A; GDF-15, growth differentiation factor-15; RELM-ß, resistin like molecule-ß; sThrombomodulin, soluble thrombomodulin; SSc-PAH, systemic sclerosis related pulmonary arterial hypertension; SSc, systemic sclerosis; IPAH, idiopathic pulmonary arterial hypertension; HV, healthy volunteer; Sens, sensitivity; PH, pulmonary hypertension; Spec, specificity; mPAP, mean pulmonary artery pressure; PVR, pulmonary vascular resistance; CI, cardiac index; RVSP, right ventricular systolic pressure; DL_CO_, diffusing capacity for carbon monoxide; sPAP, systolic pulmonary artery pressure; EC, endothelial cells*.

### N-terminal pro-brain natriuretic peptide (NT-proBNP)

NT-proBNP is a marker of myocardial stress and therefore a non-specific marker for pulmonary hypertension (PH). Brain type natriuretic peptide (BNP) and NT-proBNP remain the only blood-based biomarkers suggested by guidelines for routine clinical use (21). NT-proBNP is an inactive cleavage product released during the activation of BNP from its prohormone. BNP is released in response to ventricular stretch and stimulates natriuresis and diuresis via the kidney in order to reduce ventricular preload. NT-proBNP is elevated in PH of any cause (22) and correlates with echocardiographic, hemodynamic and functional measurements (23–25).

NT-proBNP may be elevated in systemic sclerosis in the absence of pulmonary hypertension as a result of left ventricular disease and primary myocardial involvement (26).

In a prospective observational study of 109 patients with systemic sclerosis, including 68 with PH and 41 without PH at right heart catheter, Williams et al. set out to evaluate the utility of NT-proBNP concentrations as a screening tool for PAH. NT-proBNP concentration was significantly higher in patients with PAH than without (1474 pg/ml vs. 139 pg/ml respectively, *p* = 0.0002). The authors also reported a significant correlation between NT-proBNP concentration and mean pulmonary arterial pressure (mPAP) (*r* = 0.62, *p* < 0.0001), pulmonary vascular resistance (PVR) (*r* = 0.81, *p* < 0.0001) and right atrial pressure (RAP) (*r* = 0.53, *p* < 0.0001) at right heart catheterization (RHC). For the ability to accurately diagnose PAH a threshold of 395 pg/ml was selected, returning a sensitivity 55.9%, specificity 95.1%, PPV 95.1% and NPV 56.5%. Longitudinal analysis of baseline and change in serial NT-proBNP measurements both demonstrated significant prognostic utility (27). More recent work has provided validation, with Chung et al. reporting a sensitivity and specificity of 73 and 78% respectively for NT-proBNP at a threshold of 210 pg/ml, slightly superior to that of BNP at 71 and 59% respectively at a threshold concentration of 64 pg/ml (13).

Comparing between PAH phenotypes in a study of 98 prevalent PAH patients (SSc-PAH *n* = 55; IPAH *n* = 38; Anorexigen *n* = 5), Mathai et al. found that NT-proBNP levels were significantly higher in the SSc-PAH group vs. IPAH group (1846 pg/ml vs. 808.5 pg/ml respectively, *p* < 0.01), and this was despite a significantly higher mPAP in the patients with IPAH (41 mmHg vs. 48 mmHg, SSc vs. IPAH respectively, *p* < 0.01). The authors also noted stronger correlations between NT-proBNP concentrations and hemodynamic measures of PAH for patients with SSc-PAH than for those with IPAH; cardiac index (CI) (*r* = −0.58, *p* < 0.01 vs. *r* = −0.46, *p* < 0.01 respectively); PVR (*r* = 0.54, *p* < 0.01 vs. *r* = 0.41, *p* < 0.01 respectively). When serial protein measurements were analyzed in each subgroup, the prognostic value of NT-proBNP for predicting death remained only in the group with SSc-PAH (SSc-PAH: hazard ratio (HR) 3.07, *p* < 0.01; IPAH: HR 2.02, *p* = 0.29) (14).

The DETECT study investigated a population of SSc patients who were enriched for the presence of PAH by the inclusion of patients with a DL_CO_ < 60% predicted (10). NT-proBNP was included in a final 2-step algorithm which also included electrocardiography and echocardiography to select patients to proceed to RHC. Sensitivity for the detection of PAH was high (96%) but specificity was only 48%.

Both BNP and NT-proBNP levels have been demonstrated to be important prognostic predictors at baseline in PAH (28, 29). Subsequently, the change in NT-proBNP level after therapy was shown to be a powerful independent predictor of survival (30). More recently three large studies have confirmed the importance of changes in NT-proBNP in the risk stratification of patients with PAH during follow-up. (31, 12, 32).

### Endoglin

Transforming growth factor beta (TGF-ß) signaling has been strongly implicated in the pathogenesis of PAH, and extensively studied, particularly with regard to bone morphogenetic protein receptor type-2 mutations (26). TGF-ß signaling regulates several processes including cellular proliferation and angiogenesis. Endoglin (Eng) is a transmembrane protein expressed in endothelial cells which acts as a TGF-ß signaling complex component (33).

Both TGF-ß serum concentration and Eng level are raised in IPAH patients, with Eng localized to endothelial cells in tissue samples (34). Germline Eng mutation have shown a protective effect against the development of pulmonary hypertension in heterozygous models exposed to chronic hypoxia (34).

Coral-Alvarado et al. investigated circulating Eng concentration in 60 patients (20 SSc-PAH; 20 SSc-no PAH; 20 HV). PH was diagnosed by estimation of systolic pulmonary artery pressure >35 mmHg, or tricuspid regurgitant jet velocity >3 m/s. The authors report higher Eng concentrations in the SSc-PAH group, however possibly due to small study numbers, the difference is only statistically significant between SSc-PAH vs. healthy volunteer (HV) groups (SSc-PAH: 6.89 ng/ml, SSc-no PAH: 6.2 ng/ml, HV: 5.42 ng/ml; SSc-PAH vs. SSc-no PAH *p* = 0.2447, SSc-PAH vs. HV *p* = 0.0006, SSc-no PAH vs. HV *p* = 0.057). There was no correlation noted between Eng concentration and echocardiographic measurements of PH (15).

There is some evidence for altered Eng expression in PAH, however in SSc specifically, this evidence is weak in part due to small study sizes and study design. Given the potential role of Eng in TGF-ß signaling, a role in the pathogenesis of PAH remains reasonable, however further work in this area is needed to establish its role.

### VEGF-A

Vascular Endothelial Growth Factor-A (VEGF-A) is a member of the PDGF superfamily of growth factors. It is one of the most potent regulators of angiogenesis, and acts on vascular endothelial cells through stimulation of KDR (VEGF receptor 2) and FLT-1 (VEGF receptor 1) to promote angiogenesis, increase vascular permeability and stimulate endothelial cell migration (35, 36).

Serum VEGF-A concentrations are known to be elevated in patients with PAH and have been demonstrated within plexiform lesions of remodeled vasculature (17, 37).

In a study including 53 participants (SSc-PAH *n* = 20, SSc-no PAH *n* = 20, HV *n* = 13) Papaioannou et al. examined the relationship of serum VEGF-A concentration to echocardiographic markers of pulmonary hypertension. In this study, participants were treatment naive, and any patients with pulmonary fibrosis were excluded. Estimated sPAP >35 mmHg was used to define patients with SSc-PAH. The authors found significantly higher VEGF-A concentrations in all patients with SSc as compared to HV (267 pg/ml vs. 192 pg/ml respectively, *p* < 0.01), and further found that those with SSc-PAH had higher levels than those with SSc-no PAH (352 pg/ml vs. 240 pg/ml respectively, *p* < 0.01). In patients with SSc, significant correlations were found between serum VEGF-A concentration and systolic pulmonary arterial pressure (sPAP) (*r* = 0.58, *p* < 0.01); MRC dyspnoea score (*r* = 0.34, *p* = 0.031); and DLco (*r* = −0.47, *p* < 0.01). In multivariate modeling of sPAP as the dependent variable, VEGF-A concentration remained a significant predictor when adjusted for age and gender (17).

VEGF-A expression is known to be upregulated in both patients PAH, and with systemic sclerosis, both conditions characterized by pathologically excessive endothelial activation. In patients with SSc-PAH the VEGF pathway is upregulated, however baseline levels have not been assessed for utility as diagnostic biomarkers.

### Placenta growth factor (PLGF) and soluble vascular endothelial growth factor (VEGF) receptor 1 (sFLT-1)

Placenta growth factor is a member of the vascular endothelial growth factor family of proteins which binds with high affinity for VEGF receptor 1 (FLT-1/ VEGF-R1), but not for VEGF receptor 2 (KDR/ VEGFR2)—regarded as the main effector protein of VEGF signaling (38). PlGF alone does not stimulate tyrosine kinase phosphorylation or proliferation in human endothelial cell lines, however the addition of PlGF potentiates the effect of VEGF-A in stimulating proliferation of cultured endothelial cells (38).

sFLT-1 is a variant of VEGF receptor 1 (FLT-1) which can bind VEGF-A, VEGF-B, and Placenta growth factor (PlGF). It functions as a decoy receptor, downregulating free ligand and therefore thought to control excessive endothelial activity (39).

Recognizing the need for further study into diagnostic biomarkers for patients with SSc-PAH, McMahan et al. designed a case-control study of 77 patients with SSc (37 with PH, 40 without PH). The groups were unbalanced for age (64.9 vs. 55.9 respectively, *p* < 0.01) and lung volumes (FVC% 67.5 vs. 88.1 respectively, *p* < 0.01). Diagnosis of PH was based on mPAP ≥ 25 mmHg at right heart catheterization. The authors report that both PlGF (24.8 pg/ml vs. 19.1 pg/ml, *p* = 0.02) and sFLT-1 (101.8 pg/ml vs. 89.7 pg/ml, *p* = 0.02) are significantly upregulated in patients with PH that in those without. Both proteins were significantly inversely correlated to DLco (PlGF: *r* = −0.31, *p* = 0.01 and sFLT-1: *r* = −0.29, *p* = 0.01). sFLT-1 was also correlated to RVSP (*r* = 0.32, *p* = 0.01) (16).

This study was designed to evaluate potential biomarkers of pulmonary hypertension in systemic sclerosis. No comment is made about extent of pulmonary fibrosis, so it remains conceivable that these are imbalanced given the difference in baseline pulmonary function tests, and it is not clear why no comparisons were given for protein concentration and invasive right heart catheter measurements. Although protein concentration changes are noted, no statistics have been given for the performance of these proteins as diagnostic markers.

### GDF-15

Growth Differentiation Factor-15 (GDF-15) is a member of the TGF-ß superfamily of cytokines playing an important role in cell growth and differentiation. It is a stress responsive cytokine associated with tissue damage and inflammation. Increased levels have been reported in heart failure, atherosclerosis, endothelial dysfunction and diabetes and have been linked to disease progression and prognosis (40).

In treatment naïve IPAH, serum GDF-15 is increased and is a significant predictor of survival (41). In a mixed cohort of PAH patients, tissue levels of GDF-15 are increased—localizing to the pulmonary endothelium, and in remodeled vessels strong signals are identified in plexiform lesions (42). *In-vitro* studies using pulmonary endothelial cells and varying concentrations of GDF-15 resulted in reduction in hypoxia induced apoptosis suggesting a potential pathological mechanism in PAH (42).

Meadows et al. studied a cohort of 111 patients (SSc-PAH *n* = 30, SSc-no PAH *n* = 24, IPAH *n* = 44, HV *n* = 13) for circulating GDF-15 concentrations. PH was defined at right heart catheterization. Patients with PAH were already established on PH specific therapy at the time of entry to study. Both plasma and tissue levels of GDF-15 were elevated in SSc-PAH (442 pg/ml), and differentiated it from SSc without PAH (108 pg/ml, *p* = 0.0004), IPAH (173 pg/ml, *p* = 0.0003) and HV (66 pg/ml, *p* = 0.0013). Within the SSc subgroup, GDF-15 levels correlated with echocardiographic RVSP (*r* = 0.556, *p* < 0.001), and with NT-proBNP concentration (*r* = 0.484, *p* < 0.001), but not with other invasive hemodynamics. On diagnostic ROC analysis, GDF-15 has been shown to have good discriminative power with area under curve (AUC) 0.91 for differentiation of SSc-PAH from SSc without PH with an optimal threshold for GDF-15 of 125 pg/ml demonstrating 93% sensitivity and 88% specificity for the presence of SSc-PAH. Furthermore, patients below this threshold were found to have significantly improved survival (18).

### Resistin-like molecule-ß (RELM-ß)

RELM-ß is a member of a relatively newly described resistin family. Largely studied through their effects on animal models, these proteins have been shown to induce angiogenesis and vascular remodeling (19).

Following the identification that hypoxia induced mitogenic factor(HIMF) is upregulated in animal models of PH, Angelini et al. sought to evaluate this in human tissues. In a small study involving 26 prevalent patients (SSc-PAH *n* = 9, IPAH *n* = 11, HV *n* = 6), the authors found that in human lung tissue samples, RELM-ß (a close human homolog to HIMF) is upregulated in patients with SSc-PAH as compared to healthy control (*p* < 0.01, measured by relative intensity on western blot) and localizes to remodeled vasculature. In comparison, although some expression of RELM-ß was noted in remodeled vessels of patients with IPAH, this was inconsistent, and relative quantification showed no difference between IPAH and HV concentrations. Additional *in-vitro* study showed mitogenic activity of RELM-ß on both human lung microvascular endothelial cells and human pulmonary artery smooth muscle cells (19).

This is a relatively novel candidate protein, which appears to show higher expression in SSc-PAH, however more work is needed to assess its concentration in the circulating compartment if it is to be considered further as a biomarker as lung tissue samples are not practical for this purpose.

### Soluble thrombomodulin (sThrombomodulin)

Thrombomodulin is a glycoprotein expressed on endothelial cells. Its physiological function is to bind thrombin and alter its activity, to subsequently activate protein C (15). The pathogenesis of both systemic sclerosis and PAH involves and injury to and activation of the vascular endothelium. Soluble thrombomodulin is increased in conditions associated with endothelial damage (43).

Stratton et al. studied 92 patients (SSc-PAH *n* = 34, SSc-no PAH *n* = 38, HV *n* = 20) and found that sThrombomodulin was increased plasma of patients with SSc-PAH (65.4 ng/ml) compared to SSc without PH (43.3 ng/ml, *p* < 0.05), and healthy controls (38.1 ng/ml, *p* < 0.05). There was no difference in circulating concentration between SSc without PH and healthy control (20). This is in contrast to previous studies which have shown a significant decrease in circulating sThrombomodulin concentration in patients with PAH (IPAH and PAH due to Eisenmenger's' syndrome) compared to healthy controls (26 vs. 44 ng/ml respectively, *p* = 0.0001) (44).

## Discussion

A biomarker has been defined by the NIH as “a characteristic that is objectively measured and evaluated as an indicator of normal biological processes, pathogenic processes, or pharmacologic responses to a therapeutic intervention.” Strimbu, et al. (45) NT-proBNP is the most widely studied circulating biomarker in clinical use in patients with suspected or known PAH. Elevations in NT-proBNP result from right ventricular (RV) strain as a result of increased RV afterload. As it does not reflect the underlying pathophysiology of the pulmonary arterial vasculopathy resulting in increased RV afterload in PAH, NT-proBNP levels can be elevated due to other pathophysiological processes including increased RV afterload due to PH arising from left heart disease and from disease processes directly affecting the myocardium. As such, the specificity of NT-proBNP in the diagnosis of SSc-PAH tends to be rather low resulting in a significant number of RHCs being performed in patients who do not in the end have PAH (10). Furthermore, given the dismal prognosis in SSc-PAH, identifying patients early in their disease process before the development of RV strain is desirable (46, 47). The identification of a biomarker or panel of biomarkers which more reflect the underlying pulmonary vasculopathy in SSc-PAH prior to the development of RV strain is therefore of interest.

The data described in the current manuscript summaries the current evidence for various candidate circulating diagnostic biomarkers for SSc-PAH, several of which do relate known pathways known to be important in PAH pathogenesis, especially the TGF-ß and VEGF pathways. Further study within well phenotyped cohorts of patients to compare the performance of these candidate circulating biomarkers against NT-proBNP and the DETECT protocol are clearly warranted.

## Author contributions

PH wrote and reviewed manuscript. AL supervised, and reviewed manuscript making comments and alterations. RC supervised writing and manuscript, reviewed and made significant alterations and changes.

### Conflict of interest statement

The authors declare that the research was conducted in the absence of any commercial or financial relationships that could be construed as a potential conflict of interest.
